# Evaluation of contemporary echocardiographic and histomorphology parameters in predicting mortality in patients with endomyocardial biopsy-proven cardiac AL amyloidosis

**DOI:** 10.3389/fcvm.2022.1073804

**Published:** 2023-01-24

**Authors:** Efstratios Koutroumpakis, Adam Niku, Christopher K. Black, Abdelrahman Ali, Humaira Sadaf, Juhee Song, Nicolas Palaskas, Cezar Iliescu, Jean-Bernard Durand, Syed Wamique Yusuf, Hans C. Lee, L. Maximilian Buja, Anita Deswal, Jose Banchs

**Affiliations:** ^1^Division of Internal Medicine, Department of Cardiology, The University of Texas MD Anderson Cancer Center, Houston, TX, United States; ^2^Department of Pathology and Laboratory Medicine, McGovern Medical School at The University of Texas Health Science Center at Houston, Houston, TX, United States; ^3^Department of Biostatistics, The University of Texas MD Anderson Cancer Center, Houston, TX, United States; ^4^Division of Cancer Medicine, Department of Lymphoma-Myeloma, The University of Texas MD Anderson Cancer Center, Houston, TX, United States; ^5^Division of Cardiology, University of Colorado School of Medicine, Aurora, CO, United States

**Keywords:** cardiac AL amyloidosis, echocardiography, histomorphology, pathology, mortality, left atrial strain (LA strain), stem cell transplant (SCT)

## Abstract

**Introduction:**

This study examined the role of echocardiographic and cardiac histomorphology parameters in predicting mortality in patients with cardiac AL amyloidosis.

**Methods:**

Patients with endomyocardial biopsy-proven cardiac AL amyloidosis treated at MD Anderson Cancer Center between 6/2011 and 6/2020 were identified. Stored echocardiographic images and endomyocardial biopsy samples were processed for myocardial strain analysis and a detailed histomorphology characterization.

**Results:**

Of 43 patients; 44% were women and 63% white. Median age was 65 years; 51% underwent stem cell transplantation (SCT). Thirty patients (70%) died during follow up (median follow up: 4.1 years). Lower LA strain (<13.5%) and absence of SCT as a time-varying covariate were significantly associated with increased risk of death in the multivariate cox regression analysis. Higher LV mass and lower RV tricuspid annular plane systolic excursion were associated with increased odds of having ≥5% interstitial amyloid deposition on biopsy in the multivariate logistic regression analysis.

**Conclusion:**

Lower LA strain independently predicted mortality in our cohort, and its performance in the routine assessment of AL amyloidosis may be beneficial. Furthermore, SCT for cardiac AL amyloidosis was associated with improved OS. These findings need to be confirmed by larger studies in the era of contemporary systemic therapies.

## Introduction

Immunoglobulin light chain (AL) amyloidosis involves the heart in approximately half of all patients with AL amyloidosis (1). Early diagnosis of cardiac amyloidosis is critical because the median survival after onset of heart failure has been reported as low as 6 months in patients with documented cardiac involvement (2, 3). The diagnosis of cardiac AL amyloidosis is often suggested by echocardiographic or cardiac MRI findings, but endomyocardial biopsy (EMB) is sometimes necessary for the establishment of diagnosis (4). Patients are treated with systemic therapy with or without stem cell transplantation (SCT) to achieve hematologic remission of the underlying plasma cell dyscrasia, halting the progression of end organ damage (4). However, there are currently no pharmacologic agents specific to cardiac AL amyloidosis shown to improve cardiovascular outcomes. When coupled with complete hematologic remission, heart transplantation has demonstrated some success as a treatment option with a small study demonstrating a median survival of 10.8 years after transplantation (5). Of note, complete hematologic remission is not necessary prior to heart transplantation (6).

Appropriate patient risk stratification is essential in guiding treatment of the underlying plasma cell dyscrasias. The Mayo 2004, Mayo 2004 with European modification, Mayo 2012 and Boston University staging systems are recognized as well validated systems to predict survival in general in patients with AL amyloidosis (7–10). These staging systems use troponin levels (T or I), and natriuretic peptide levels with or without free light chain differences to stratify patients for overall survival (8). In some recent reports, the prognostic value of these systems has been augmented with echo-derived strain patterns, such as left ventricular (LV) global longitudinal strain (GLS) and the apical to basal longitudinal strain gradient (11). Furthermore, the capillary density within the myocardium on histopathology has also been shown to improve the prognostic value of the 2012 Mayo staging system (12).

Although EMB carries a sensitivity that approaches 100% for the diagnosis of cardiac AL amyloidosis, the value of a detailed histomorphology analysis in predicting mortality is not well studied (13). Furthermore, the correlation of echocardiographic findings with histomorphology characteristics has not been thoroughly examined. The aim of this study was to assess the correlation of echocardiographic parameters with histomorphology findings, and their association with all-cause mortality.

## Materials and methods

### Patient selection and data collection

Adult patients (age 18 years or older) diagnosed with cardiac AL amyloidosis by endomyocardial biopsy at the University of Texas MD Anderson Cancer Center between the dates 6/2/2011 to 6/16/2020 were identified. Among those, patients who had adequate endomyocardial biopsy samples stored for detailed histomorphology analysis were selected.

**FIGURE 1:**
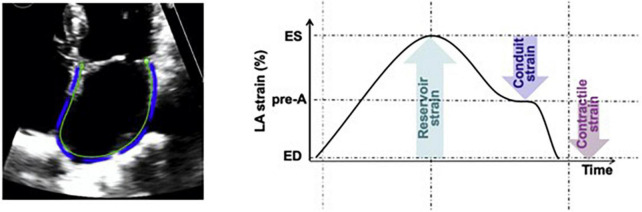
Measurement of reservoir LA strain as [(end systole LA strain) – (end diastole LA strain)] using LA-focused apical four chamber views. Reprinted from Singh et al. (15) with permission from Elsevier.

Patient electronic medical records were manually reviewed for demographic characteristics, clinical, laboratory and echocardiographic data. Age, gender, race, cardiovascular comorbidities and therapies targeting the underlying plasma cell dyscrasia were collected. Additionally, laboratory parameters including hemoglobin, platelets, creatinine, serum free light chains and cardiac biomarkers as well as routine 2D and Doppler echocardiographic parameters were abstracted. All echocardiographic images were captured using GE echocardiography machines. Laboratory values and echocardiograms within a 2-month period prior to the date of endomyocardial biopsy were available for >95% of patients. With regards to cardiac biomarkers, most patients had B-type natriuretic peptides (BNP 76% vs NT-proBNP 24%) and traditional troponin I (66% vs high sensitivity troponin T 34%) levels. Stored echocardiographic images were further processed for longitudinal (global, apical, mid, basal), radial and circumferential strain of the LV, right ventricle (RV) free wall strain, left atrium (LA) reservoir strain and right atrium (RA) strain. Strain measurements were performed by a physician board certified in echocardiography and blinded to the clinical outcomes. Semiautomated strain analysis was performed using a vendor independent software (Epsilon EchoInsight Software) (14). Strain analysis of the LA and RA was performed on zoomed LA and RA-focused apical four-chamber views when available or the standard apical four-chamber views when LA and RA-focused views of sufficient quality were not available. For the purposes of this study, LA strain refers to reservoir LA strain, calculated as end-systole LA minus end-diastole LA strain (Figure 1) (15). The subcomponents of LA strain including conduit and active emptying LA strain were not calculated in this study. The terms “LA strain” and “reservoir LA strain” are used interchangeably in this manuscript.

### Histomorphology analysis

**FIGURE 2:**
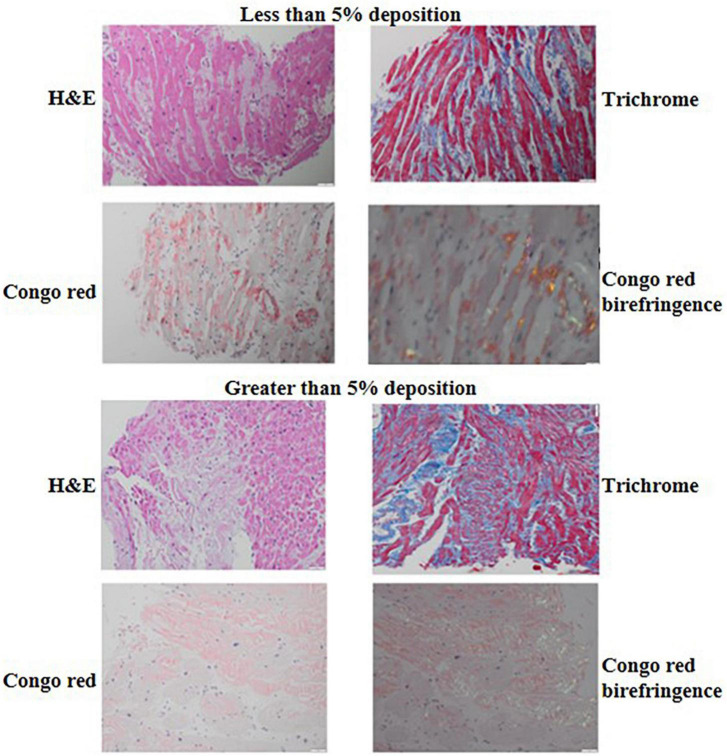
H&E, trichrome, Congo red and Congo red birefringence appearance of a biopsy sample with <5% **(top)** compared to a biopsy sample with ≥5% interstitial amyloid deposition **(bottom)**. The H&E and trichrome photomicrographs were captured using the 20× lens on 50 μm scale while the Congo red and Congo red birefringence photomicrographs were captured using the 40× lens on 20 um scale.

Formalin-fixed and paraffin-embedded tissue samples obtained by endomyocardial biopsy were used. Amyloid was detected using H&E and Congo red staining of tissue sections reviewed with light microscopy and Congo red staining, polarized light, showing green-yellow-orange birefringence. Immunohistochemical classification of the extent of amyloid and pattern of deposition was performed using the Larsen BT et al. scoring by two cardiovascular pathologists (13). The extent of interstitial and vascular amyloid deposition was evaluated semi quantitatively by light microscopy. The extent of interstitial deposition of amyloid was graded based on the percentage of myocardial area involved as follows: 0: no interstitial deposits, 1+: interstitial deposits involving 1 to <5% of myocardial area, 2+: interstitial deposits involving 5 to <50% of myocardial area, and 3+: interstitial deposits involving >50% of myocardial area (Figure 2). Four patterns of interstitial deposition were recorded: (i) a diffuse pericellular pattern, characterized by broad zones of uniform lace-like deposition around individual cardiomyocytes; (ii) a discrete pericellular pattern, characterized by micronodular areas of pericellular deposition (generally < 20 myocytes) interspersed with uninvolved myocardium; (iii) a nodular pattern, characterized by solid deposits with interstitial architectural effacement; and a (iv) mixed pattern. Vascular deposition of amyloid was graded as (i) non-obstructive (<75% reduction in the luminal cross-sectional area), or (ii) obstructive (≥75% reduction in the luminal cross-sectional area). Three patterns of vascular deposition were recorded: (i) an arterial pattern; (ii) a venous pattern; and (iii) a mixed pattern. Endocardial involvement, cardiomyocyte degeneration and presence of fibrosis were also assessed and recorded. The cardiovascular pathologists were blinded to the clinical outcomes of the patients.

### Clinical outcomes

The primary clinical outcome of the study was all-cause mortality. The electronic medical records of the patients were reviewed to determine mortality, date of death or date of last follow up. If unavailable in the chart, mortality data was supplemented by search of online obituaries. Associations of echocardiographic parameters with histomorphology characteristics and all-cause mortality were examined. Furthermore, the Mayo 2004, Mayo 2012 and Boston University staging systems were used to stratify the patients in our cohort based on available cardiac enzyme and light chain levels. Based on the Mayo 2004 staging system we used the cutoff values of NT-proBNP >332 ng/L, cardiac troponin T > 0.035 μg/L, and cardiac troponin I > 0.1 μg/L to group the patients of our cohort into stage I (all cardiac enzyme levels below the cutoff level), II (one cardiac enzyme level elevated) or III (both cardiac enzymes elevated) (7). Patients who only had BNP levels available but no NT-proBNP levels were not classified based on the Mayo 2004 staging system. Based on the Mayo 2012 staging system, the patients in our cohort were assigned a score of 1 for each of: (i) free light chain difference (FLC-diff) ≥ 18 mg/dL, cardiac troponin T ≥ 0.025 ng/mL, and NT-ProBNP ≥ 1,800 pg/mL, creating stages I to IV with scores of 0–3 points, respectively (8). Patients who only had BNP and cardiac troponin I levels available but no NT-proBNP or cardiac troponin T levels were not classified based on the Mayo 2012 staging system. Based on the Boston University staging system, the patients in our cohort were grouped into stage I if troponin I was ≤ 0.1 ng/mL and BNP ≤81 pg/mL, stage II if troponin I was >0.1 ng/mL OR BNP > 81 pg/mL, stage III if troponin I was > 0.1 ng/mL AND BNP > 81 pg/mL and stage IV if troponin I was >0.1 ng/mL AND BNP > 700 pg/mL (10). Patients who only had NT-proBNP and cardiac troponin T levels available, but no BNP or cardiac troponin I levels were not classified based on the Boston University staging system. Associations between the different stages of each grading system with mortality were examined.

### Statistical analysis

Continuous variables are presented either as mean values ± standard deviation (SD) or median values and interquartile range (IQR), based on normality of distribution. Categorical variables are presented as frequencies and percentages. The association between each covariate on echo findings (presence of interstitial deposit ≥ 5% or not) was assessed by univariate and multivariate logistic regression models (selected by stepwise selection if possible).

Overall survival (OS) is calculated as time interval from endomyocardial biopsy to death. Those who survived were censored at the time of last follow-up. Univariate and multivariate Cox regression models were fitted to evaluate the effect of each covariate on death. A multivariate Cox regression model initially included all covariates with significant *p*-values based on univariate models and selected by stepwise selection method. We tested the proportionality of hazards with the use of time-varying covariates. SCT was performed before or after biopsy. We considered SCT as a time-varying covariate to take into account the effect of variable timing of SCT after biopsy on mortality. Kaplan-Meier plots were produced for covariates that were significantly associated with OS based on Log-rank testing. Simon-Makuch plots was produced for time-varying covariate. A *p*-value less than 0.05 was used to assess statistical significance. SAS 9.4 (SAS Institute Inc., Cary, NC, USA) was used for data analysis.

### Ethical approval

This study was approved by the institutional review board (2022-0614).

## Results

### Patient population

A total of 43 patients diagnosed with cardiac AL amyloidosis by endomyocardial biopsy were identified. Median age was 65.4 years (IQR 59.2, 70.5); 44% of the patients were women and 63% were white. Hypertension was present in 70%, dyslipidemia in 40% and diabetes mellitus in 16% of the patients. Five patients (12%) had a prior history of clinical coronary artery disease. Multiple myeloma was diagnosed in 44% of the total cohort while MGUS in 35%. Out of the 19 patients with multiple myeloma, 12 (63%) met CRAB criteria while the remaining 7 patients (37%) had smoldering myeloma with amyloidosis. For the treatment of their underlying plasma cell dyscrasia, 72% of patients received a proteasome inhibitor, 56% an alkylating agent, 30% an immunomodulatory drug and 9% an anti-CD38 monoclonal antibody. A total of 22 patients (51%) underwent autologous SCT. The reason that the rest 21 patients did not undergo SCT, included multiorgan failure upon diagnosis/late diagnosis in 19, financial reasons in 1 and social reasons (lack of caregiver) in 1 patient. The median BNP of the patients in our cohort was 472 pg/mL (IQR 163, 1409) and median troponin I was 0.07 ng/mL (IQR 0.04, 0.24). When using institutional assay limits of normal, a total of 89% (*n* = 34/38) of patients had elevated natriuretic peptide levels, while 77% (25/31) had elevated troponin levels. Mean ± SD hemoglobin was 11.6 ± 1.9 g/dL, median creatinine 0.95 mg/dL (IQR 0.82, 1.52) and median platelet count 241 K/μL (IQR 165, 306). Additional data on patient demographics and baseline clinical characteristics are presented in Table 1 and Supplementary Table 1.

**TABLE 1:** Demographic and baseline clinical characteristics of 43 patients with endomyocardial biopsy-proven cardiac AL amyloidosis.

	Total *N* = 43
Age at biopsy in years, median (IQR)	65 (59,70)
Weight in kg, mean ± SD	85 ± 20
Race, *n* (%):	
– White	27 (62.8)
– Black	12 (27.9)
– Other	4 (9.3)
Female sex, *n* (%)	19 (44.2)
Hypertension, *n* (%)	30 (69.8)
Dyslipidemia, *n* (%)	17 (39.5)
Diabetes mellitus, *n* (%)	7 (16.3)
Coronary artery disease, *n* (%)	5 (11.6)
End-stage renal disease on hemodialysis, *n* (%)	3 (7)
Peripheral arterial disease, *n* (%)	2 (4.7)
Multiple myeloma, *n* (%)	19 (44.2)
MGUS, *n* (%)	15 (34.9)
Leukemia/lymphoma, *n* (%)	3 (7.0)
Waldenstrom’s macroglobulinemia, *n* (%)	3 (7.0)
**Plasma cell dyscrasia therapies:**	
Stem cell transplantation (SCT), *n* (%)	22 (51.2)
SCT before endomyocardial biopsy, *n* (%)	5 (11.6%)
Proteasome inhibitors (e.g., bortezomib), *n* (%)	31 (72.1)
Alkylating agents (e.g., cyclophosphamide), *n* (%)	24 (55.8)
Immunomodulatory drugs (e.g., lenalidomide), *n* (%)	13 (30.2)
Anti-CD38 monoclonal Abs (e.g., daratumumab), *n* (%)	4 (9.3)
Rituximab, *n* (%)	4 (9.3)
Elotuzumab, *n* (%)	2 (4.7)
Vorinostat, *n* (%)	1 (2.3)
**Baseline laboratory values:**	
Hemoglobin < 12 g/dL, *n* (%)	21 (55.3)
Platelets < 150 K/uL, *n* (%)	8 (19)
Absolute Neutrophils < 1.7 × 1,000/mm^3^, *n* (%)	2 (5.6)
INR > 1.1, *n* (%)	21 (61.8)
Creatinine in mg/dL, median (IQR)	0.95 (0.82,1.52)
Elevated natriuretic peptide levels (BNP or NT-proBNP), *n* (%)	34 (89.5)
Elevated troponin levels (I or T), *n* (%)	24 (77.4)
**Mayo 2004 staging, *n* (%):**	
Unknown staging	28
Stage II	3 (20)
Stage III	12 (80)
**Mayo 2012 staging, *n* (%):**	
Unknown staging	30
Stage I	1 (7.7)
Stage II	1 (7.7)
Stage III	7 (53.8)
Stage IV	4 (30.8)
**Boston University staging, *n* (%):**	
Unknown staging	21
Stage I	2 (9.1)
Stage II	7 (31.8)
Stage IIIA	6 (27.3)
Stage IIIB	7 (31.8)
Composite staging, *n* (%): *	
Unknown Staging	9
Early stages (I-II by Mayo or Boston)	10 (29.4%)
Advanced stages (III–IV by Mayo or Boston)	24 (70.6%)

*Using Mayo 2004, Mayo 2012 and Boston University staging systems; if at least one of staging is indicated III or IV then it was considered high; if at least one staging was performed and all staging indicated I or II, then considered low.

### Echocardiographic characteristics

Mean LV ejection fraction (LVEF) ± SD of the cohort was 52.4 ± 10.3%, while 42% of the patients had an LVEF of <50%. Most patients had impaired LV relaxation with 94% having a lateral e’ velocity of <10 cm/sec and 81% a septal e’ velocity of <7 cm/sec. Left atrial volume index (LAVI) was >34 mL in 49% and E/e’ average was >14 in 48% of patients. Most patients in our cohort also had evidence of RV dysfunction with 76% having a tricuspid annular plane systolic excursion (TAPSE) of <1.6 cm. The mean LV GLS ± SD of the cohort was –11.7 ± 3.0%, while all patients had an abnormal LV GLS [>–18%, range (–17.27, –6.86)]. Mean LV apical longitudinal strain ± SD was –15.6 ± 4.6% while mean mid LV longitudinal strain ± SD was –11.1 ± 3.4 and median basal LV longitudinal strain –7.2 (IQR: –10.3, –4.8). A detailed list of echocardiographic measurements is presented in Table 2.

**TABLE 2:** Echocardiographic and histomorphology characteristics of 43 patients with endomyocardial biopsy-proven cardiac AL amyloidosis.

Echocardiographic characteristics:	
LVEF in %, mean ± SD	52.38 ± 10.26
LVEF < 50%, *n* (%)	16 (42.1)
LVEDVi in mL/m^2^, mean ± SD	53.25 ± 17.05
LVIDD in cm, mean ± SD	4.30 ± 0.69
LVPWD in cm, median (IQR)	1.3 (1.18,1.5)
RWT > 0.42, *n* (%)	34 (87.2)
LV mass in g/m^2^, mean ± SD	203.80 ± 55.88
Lateral e’ velocity < 10 cm/s, *n* (%)	30 (93.8%)
Septal e’ velocity < 7 cm/s, *n* (%)	26 (81.3)
E/e’ average ≥ 14, *n* (%)	15 (48.4)
LAVI ≥34 mL/m^2^, *n* (%)	17 (48.6)
RV FAC < 35%, *n* (%)	16 (43.2)
RV GLS in %, median (IQR)	–17 (–21, –12)
RV S’ < 9.5 cm/s, *n* (%)	32 (86.5)
RV TAPSE < 1.6 cm, *n* (%)	28 (75.7)
LV GLS in %, mean ± SD	–11.67 ± 3.03
LV GLS ≥–18%, *n* (%)	39 (100)
Apex GLS in %, mean ± SD	–15.60 ± 4.57
Mid LV GLS in %, mean ± SD	–11.05 ± 3.41
Basal LV GLS in %, median (IQR)	–7.16 (–10.26, -4.79)
Circumferential LV strain in %, mean ± SD	-18.45 ± 5.66
Circumferential LV strain > –23%, *n* (%)	29 (76.3)
Radial LV strain in %, mean ± SD	12.11 ± 8.69
Rad LV strain < 21%, *n* (%)	33 (86.8)
LA strain > 13.5, *n* (%)	19 (50)
RA strain > 14.5, *n* (%)	19 (50)
**Histomorphology characteristics:**	
Interstitial deposit ≥5% of myocardial area, *n* (%)	26 (60.5)
Pattern of interstitial deposit, *n* (%)	
– Diffuse pericellular	23 (53.5)
– Discrete pericellular, nodular, mixed	12 (27.9)
Vascular deposit, *n* (%)	12 (27.9)
Endocardial deposit, *n* (%)	5 (11.6)
Cardiomyocyte degeneration, *n* (%)	12 (27.9)
Fibrosis, *n* (%)	9 (20.9)

### Histomorphology characterization

Out of a total of 43 endomyocardial tissue samples graded (Table 3), 35 (81%) had interstitial deposits (21% 1+, 44% 2+, 16% 3+ deposits) while 19% had no interstitial deposits. Of those with interstitial deposits, the most prevalent pattern of interstitial deposition was a diffuse pericellular pattern, present in 66% of patients, followed by the other 3 patterns equally, discrete pericellular (11%), nodular (11%), and mixed (11%). Vascular deposition of amyloid was present in 12 samples (28%); 75% of which had evidence of obstructive vascular deposition. Of those samples with vascular deposition, arterial pattern was seen in 83%, venous pattern in 8% and a mixed pattern in 8%. Cardiomyocyte degeneration was noted in 28%, fibrosis in 21% and endocardial involvement in 12% of samples (Table 2).

**TABLE 3:** Immunohistochemical classification of cardiac amyloid extent and pattern.

Extent of amyloid deposits
I. Interstitial deposits: 0; 1+ (< 5% of myocardial area); 2+ (>/= 5% but < 50% of myocardial area); 3+ (> 50% of myocardial area)
II. Vascular deposits: Obstructive or Non-obstructive
**Pattern of interstitial amyloid deposits**
I. Diffuse pericellular pattern, characterized by broad zones of uniform lace-like deposition around individual CMC
II. Discrete pericellular pattern, characterized by micronodular areas of pericellular deposition (generally < 20 CMC)
III. Nodular pattern, characterized by solid deposits with interstitial architectural effacement
IV. Mixed patterns
**Pattern of vascular amyloid deposits**
I. Arterial
II. Venous
**Endocardial amyloid deposits**
I. Present
II. Absent

Adapted from Larsen et al. (13).

### Mortality

A total of 30 patients (70%) died during the follow up of the study, and most deaths (*n* = 16; 53%) occurred within the first year from the date of endomyocardial biopsy. Median time to last follow up or death was 4.1 years (95% CI, 2.9–8.6). Median overall survival (OS) was 18.4 months (95% CI, 8.1–37).

**FIGURE 3:**
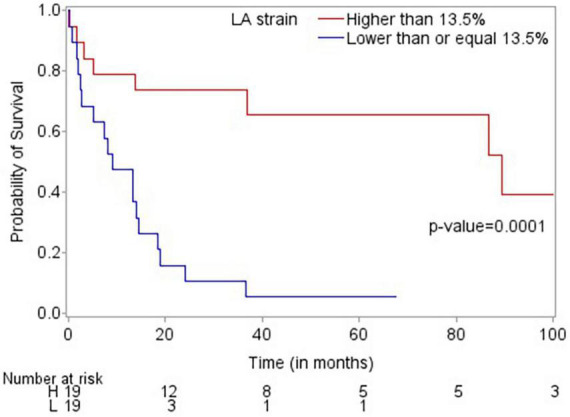
Kaplan-Meier plot demonstrating that LA strain >13.5 is associated with improved overall survival.

**FIGURE 4:**
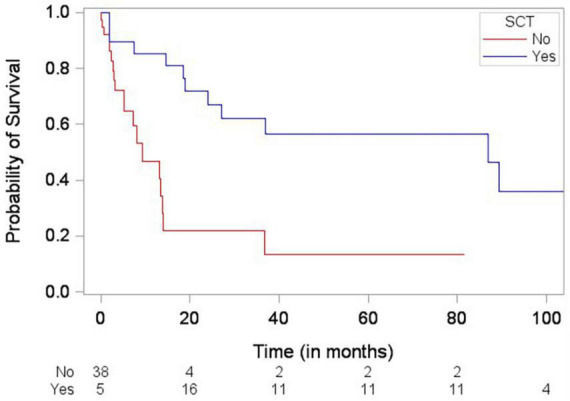
Simon and Makuch plot demonstrating stem cell transplantation (SCT) being significantly associated with decreased risk of death (*p*-value = 0.0012) based on Cox regression analysis with SCT as a time-varying covariate.

Patients who died were more likely to have a history of dyslipidemia, a baseline neutrophil count <1.7 × 1,000/mm^3^, a higher troponin I, a lower LVEF, less negative mid-LV GLS, lower RV TAPSE, lower RA strain, lower LA strain, and lower LV radial strain. Further, they were less likely to have undergone SCT (Tables 4, 5, and Supplementary Table 2). In the multivariate cox regression analysis, only lower LA strain (HR 3.8, 95% CI 1.4–10.2 for LA strain ≤13.5 vs > 13.5, *p* = 0.008) and absence of SCT as a time-varying covariate (HR 0.20, 0.06–0.65 for SCT vs no SCT, *p* = 0.007) were significant independent predictors of an increased risk of death (Figures 3, 4). Most patients underwent SCT after their endomyocardial biopsy with median time from biopsy of 76 days (17/22 who underwent SCT, 77%). Most patients who did not undergo SCT (90%) had multiorgan failure upon diagnosis. None of the histomorphology parameters was significantly associated with the risk of mortality.

**TABLE 4:** Clinical and echocardiographic characteristics significantly associated with likelihood of death in the univariate Cox regression analysis.

	Univariate Cox regression on mortality (mortality event = 29) HR (95% CI)	*p*-value
Dyslipidemia	2.660 (1.252–5.649)	0.0109
SCT*^,∧^	0.213 (0.083–0.547)	0.0012
Rituximab	3.023 (1.003–9.110)	0.0494
Absolute Neutrophils < 1.7 × 1,000/mm^3^	5.353 (1.102–26.002)	0.0375
Troponin I in ng/mL	8.379 (1.127–62.301)	0.0378
LVEF in %	0.958 (0.923–0.995)	0.0257
RV TAPSE in cm	0.299 (0.105–0.855)	0.0242
Mid LV GLS in %	1.141 (1.015–1.282)	0.0273
Radial LV strain in %	0.935 (0.884–0.989)	0.0193
LA strain ≤ 13.5%^∧^	5.394 (2.072–14.041)	0.0006
RA strain ≤ 14.5%	2.711 (1.209–6.080)	0.0155

*SCT as a time-varying covariate. ^∧^Remained statistically significant in the multivariate Cox regression survival analysis.

**TABLE 5:** Comparison of demographic, clinical, echocardiographic and histopathology characteristics of patients with cardiac AL amyloidosis who survived vs those who died during follow up.

	Survived (*n* = 13)	Died (*n* = 30)	*p*-value
Age at biopsy in years, median (IQR)	63.71 (59.23, 66.41)	66.48 (60.7, 70.49)	0.5697
Weight in kg, mean ± SD	87.25 ± 22.87	84.68 ± 19.04	0.7040
White Race, *n* (%)	9 (69.2%)	18 (60%)	0.7349
Female sex, *n* (%)	3 (23.1%)	16 (53.3%)	0.0665
Hypertension, *n* (%)	10 (76.9%)	20 (66.7%)	0.7203
**Dyslipidemia, *n* (%)**	**2 (15.4%)**	**15 (50%)**	**0.0330**
Diabetes mellitus, *n* (%)	1 (7.7%)	6 (20%)	0.4118
Coronary artery disease, *n* (%)	2 (15.4%)	3 (10%)	0.6299
**SCT, *n* (%)**	**11 (84.6%)**	**11 (36.7%)**	**0.0068**
Rituximab, *n* (%)	0 (0%)	4 (13.3%)	0.2974
Absolute neutrophils < 1.7 × 1,000/mm^3^, *n* (%)	0 (0%)	2 (7.4%)	1.0000
Troponin I in ng/mL, median (IQR)	0.04 (0.03,0.1)	0.11 (0.05,0.26)	0.0834
**LVEF in %, mean ± SD**	**57.92 ± 5.57**	**50.12 ± 10.93**	**0.0065**
LV mass > 195.7g, *n* (%)	5 (45.5%)	14 (50%)	0.7983
**RV TAPSE in cm, mean ± SD**	**1.61 ± 0.47**	**1.22 ± 0.32**	**0.0059**
RV TAPSE < 1.6 cm, *n* (%)	6 (54.5%)	22 (84.6%)	0.0912
**Mid LV GLS in %, mean ± SD**	**–13.46 ± 2.36**	**–10.10 ± 3.32**	**0.0042**
**Radial LV strain in %, mean ± SD**	**18.00 ± 10.20**	**9.70 ± 6.83**	**0.0058**
**LA strain ≤ 13.5%, *n* (%)**	**1 (9.1%)**	**18 (66.7%)**	**0.0013**
**RA strain ≤ 14.5%, *n* (%)**	**2 (18.2%)**	**17 (63%)**	**0.0123**
Interstitial deposit ≥ 5% of myocardial area, *n* (%)	9 (69.2%)	17 (56.7%)	0.4390

Statistically significant variables are marked in bold.

### Evaluation of Mayo 2004, Mayo 2012 and Boston University staging systems in predicting mortality

Based on Mayo 2004 staging system (*N* = 15), 20% of patients were in stage II and 80% in stage III. Based on Mayo 2012 staging system (*N* = 13), 7.7% of patients were in stage I, 7.7% in stage II, 53.8% in stage III, and 30.8% in stage IV. Based on Boston University staging system (*N* = 22), 9.1% were in stage I, 31.8% stage II, 27.3% stage IIIA and 31.8% stage IIIB (Table 1). Since some patients could not be staged by all the systems due to lack of complete laboratory data (presence of BNP but not NT-proBNP), we elected to use a composite staging system (*N* = 34) based on which, 29.4% of patients were in early stages (one of Mayo stages or Boston University stage was determined, but neither was III–IV) and 70.6% in advanced stages (III–IV by Mayo or Boston). When the above staging systems were tested in predicting mortality, no significant differences between early and advanced stages were identified (Table 4).

### Correlation of echocardiographic parameters with histomorphology characteristics

Lower RV TAPSE correlated significantly with interstitial amyloid deposition of ≥ 5% of the myocardial area examined on biopsy specimens in both the univariate and multivariate logistic regression analysis (OR 8.1, 95% CI 1.2–54.3 for RV TAPSE < 1.6 vs ≥ 1.6 cm, *p* = 0.032) (Supplementary Table 3). In the multivariate logistic regression analysis, higher LV mass was also associated with increased odds of having ≥ 5% of interstitial amyloid deposition on biopsy (OR 5.5, 95% CI 1.1–26.8 for > 195.7 vs ≤ 195.7 g, *p* = 0.036). No significant correlation was identified between echocardiographic measurements and other histomorphology characteristics.

## Discussion

Our study examined the role of contemporary echocardiographic and histomorphology characteristics in predicting mortality of patients with cardiac AL amyloidosis who underwent endomyocardial biopsy. Our key findings were: (1) Lower LA strain (≤13.5%) was an independent predictor of mortality in our cohort; (2) SCT for cardiac AL amyloidosis was associated with improved OS; and (3) Although LV mass and RV TAPSE were associated with higher interstitial amyloid deposition, histomorphology parameters were not significantly associated with mortality in this cohort.

Larsen et al. examined autopsy samples of patients with cardiac amyloidosis and compared several histomorphology characteristics of endomyocardial samples of AL versus ATTR amyloidosis (13). Whether this detailed histomorphology characterization has prognostic value in predicting mortality and whether any histomorphology findings correlate with echocardiographic parameters has not been extensively studied. In a retrospective study by Kristen AV et al., including patients with cardiac AL and ATTR amyloidosis (107 with cardiac AL amyloidosis), amyloid load on EMB samples was quantified as the area of immunohistochemically stained versus non-stained pixels using a special scanner (ImageJ version 1.47v). The authors of that study concluded that amyloid load was independently associated with mortality (16). In another study by Kim D. et al., amyloid load was similarly quantified as the ratio of the immunohistochemically stained area to total tissue area using a different software (InForm version 2.4.0, PerkinElmer, Waltham, MA, USA). The investigators of this study also quantified capillary density on the EMB samples as the number of capillaries, which were counted after they were stained with a specific antibody against endothelium (CD31), divided by area (mm^2^). The authors reported that lower capillary density correlated with amyloid load and predicted mortality among 67 patients with biopsy-proven AL cardiac amyloidosis (12). When added to the 2012 Mayo staging system, capillary density led to better discrimination and reclassification ability compared to the 2012 Mayo staging system alone (12). However, neither of these two studies examined other histologic characteristics such as extent and pattern of vascular deposits, cardiomyocyte degeneration, endomyocardial deposition or fibrosis. In a recent study by Pucci A. et al., it was not just the extent of amyloid deposits but the combination of amyloid deposits and fibrosis that correlated better with myocardial tissue characterization on cardiac magnetic resonance (CMR) imaging and with levels of cardiac biomarkers (17). Our study included a detailed histomorphology characterization including extent and pattern of interstitial amyloid deposits and vascular deposits, cardiomyocyte degeneration, endomyocardial deposition or fibrosis. The extent of interstitial amyloid deposition in our study was evaluated semiquantitative by light microscopy and grouped into 3 categories (<5%, 5–49%, and ≥ 50% of myocardial area). Further, our study did not assess capillary density but assessed extent and pattern of vascular deposits of amyloid. In our study, the echocardiographic assessment of LV mass and RV function (TAPSE) correlated with higher interstitial amyloid deposition; however, none of the histomorphology parameters studied, including the extent and pattern of interstitial deposition and the extent and pattern of vascular deposition, were significantly associated with mortality. Although semiquantitative analysis of amyloid deposition may be less labor intense and does not require advanced equipment compared to the quantitative analysis described in the studies above, it did not predict mortality in our cohort. This might be related to the small sample size of our study and the lower statistical power associated with an ordinary variable (semiquantitative analysis) as opposed to a continuous variable (quantitative analysis). It is also possible that the software used for the quantitative analysis in the studies of Kristen AV et al. and Kim D et al. detected histopathology changes that were missed in our semiquantitative analysis of amyloid extent.

Echocardiographic findings have been shown to predict mortality in patients with cardiac AL amyloidosis. In one of the few prospective studies published, Koyama and Falk examined 119 consecutive patients with biopsy-proven AL amyloidosis and reported that the mean LV basal strain was the only independent predictor of both cardiac and overall deaths in their multivariate analysis (18). In another retrospective study, among 42 patients with cardiac AL amyloidosis, E/e’, LV GLS and global area strain were significantly associated with death in the univariate analysis with LV GLS remaining an independent predictor of mortality in the multivariate analysis (19). In our study, lower LA strain (≤13.5%) was independently and significantly associated with an almost four times higher likelihood of mortality. This finding suggests that the routine performance of contemporary echocardiographic parameters such as LA strain in the assessment of patients with cardiac AL amyloidosis may be beneficial, and this may need to be further assessed in larger studies.

In our cohort, cardiac biomarkers and free light chains, alone or incorporated in the routinely used staging systems of Mayo 2004, Mayo 2012 and Boston University were not independently associated with increased likelihood of mortality. Although this may be due to the small size of our cohort, it highlights the issue of prognostic ability of scores in individual patients, as well as the fact that the scores are developed in all patients with systemic AL amyloidosis, who may or may not have cardiac involvement and not just in patients with cardiac amyloidosis where almost all patients have elevations in the cardiac biomarkers. The addition of contemporary echocardiographic parameters, such as LA strain, to routinely used staging models may increase their ability to predict mortality specifically in patients with cardiac amyloidosis and should be further examined in larger, prospective cohorts.

SCT as a time-varying covariate was associated with a lower likelihood of death in our cohort. However, this finding is likely related to the fact that the patients who underwent SCT were healthier at baseline (most patients who did not undergo SCT had multiorgan failure upon diagnosis). Furthermore, use of contemporary systemic therapies such as anti-CD38 monoclonal antibodies was infrequent in our cohort. Larger prospective studies are needed to further evaluate this observation in the era of contemporary systemic therapies.

None of the pharmacologic therapies used against AL amyloidosis was associated with increased or decreased likelihood of death in our analysis (although there was a trend toward increased likelihood of death in patients treated with rituximab in the univariate analysis, the small number of patients treated with rituximab does not allow for a confident conclusion. It is more likely that patients treated with rituximab were sicker at baseline than rituximab being associated with increased likelihood of death. In the multivariate analysis, rituximab was not associated with increased or decreased likelihood of death). Of note, the use of anti-CD38 monoclonal antibodies (daratumumab) was infrequent (9%) in this cohort of patients who were treated between 2011 and 2020. Therefore, the possibility that daratumumab is associated with improved likelihood of overall survival in patients with cardiac AL amyloidosis cannot be excluded based on the findings of our study.

The strengths of our study include the thorough echocardiographic and histomorphology characterization of patients with cardiac AL amyloidosis and the long follow up period (over 4 years). All patients in our study had endomyocardial biopsy-proven AL amyloidosis. Both echocardiographic and histomorphology characterization were performed blinded to mortality outcomes. Despite its strengths, our study also has limitations. The small sample size and the retrospective nature of the study with its inherent risk for bias are the two main ones. Additionally, there was variability in the timing between EMB and treatment initiation for the patients in this cohort, which may have had an impact on OS. Finally, although most of the routinely performed conventional echocardiographic parameters were included in our analysis, others, such as RV free wall thickness, LA septal thickness or valve thickness, could not be reliably measured and therefore were not included.

In conclusion, contemporary echocardiographic parameters and more specifically LA strain may have the potential to predict the risk of death in patients with cardiac AL amyloidosis, beyond the traditional laboratory and 2D echocardiographic findings. Therefore, its performance in the routine assessment of patients with suspected or confirmed cardiac AL amyloidosis and its possible incorporation into the routinely used staging systems for patients with cardiac amyloidosis may be justified and needs to be further evaluated. Although LV mass and RV TAPSE were associated with higher interstitial amyloid deposition, histomorphology parameters were not significantly associated with mortality in this cohort.

## Data availability statement

The original contributions presented in this study are included in the article/Supplementary material, further inquiries can be directed to the corresponding author.

## Ethics statement

The studies involving human participants were reviewed and approved by University of Texas MD Anderson Cancer Center Institutional Review Board. Written informed consent for participation was not required for this study in accordance with the national legislation and the institutional requirements.

## Author contributions

EK, AN, CB, and AA collected the data. HS and LB performed the histomorphology analysis. AN and JB performed the strain analysis. EK drafted the manuscript. AD and JB oversaw the project processes. All authors contributed to the data analysis interpretation and editing of the manuscript and have reviewed the final version of the manuscript and agreed with its submission.

## References

[1] Bianchi G Zhang Y Comenzo R. AL amyloidosis: current chemotherapy and immune therapy treatment strategies: JACC: cardiooncology state-of-the-art review. *JACC CardioOncol.* (2021) 3:467–87. 10.1016/j.jaccao.2021.09.003 34729520 PMC8543128

[2] Falk R Alexander K Liao R Dorbala S. AL (Light-Chain) cardiac amyloidosis: a review of diagnosis and therapy. *J Am Coll Cardiol.* (2016) 68:1323–41. 10.1016/j.jacc.2016.06.053 27634125

[3] Kyle R Linos A Beard C Linke R Gertz M O’Fallon W Incidence and natural history of primary systemic amyloidosis in Olmsted County, Minnesota, 1950 through 1989. *Blood.* (1992) 79:1817–22. 1558973

[4] Witteles R Liedtke M. AL amyloidosis for the cardiologist and oncologist: epidemiology, diagnosis, and management. *JACC Cardiooncol.* (2019) 1:117–30. 10.1016/j.jaccao.2019.08.002 34396169 PMC8352106

[5] Grogan M Gertz M McCurdy A Roeker L Kyle R Kushwaha S Long term outcomes of cardiac transplant for immunoglobulin light chain amyloidosis: the mayo clinic experience. *World J Transplant.* (2016) 6:380–8. 10.5500/wjt.v6.i2.380 27358783 PMC4919742

[6] Estep J Bhimaraj A Cordero-Reyes A Bruckner B Loebe M Torre-Amione G. Heart transplantation and end-stage cardiac amyloidosis: a review and approach to evaluation and management. *Methodist Debakey Cardiovasc J.* (2012) 8:8–16. 23227279 10.14797/mdcj-8-3-8 PMC3487570

[7] Dispenzieri A Gertz M Kyle R Lacy M Burritt M Therneau T Serum cardiac troponins and N-terminal pro-brain natriuretic peptide: a staging system for primary systemic amyloidosis. *J Clin Oncol.* (2004) 22:3751–7. 10.1200/JCO.2004.03.029 15365071

[8] Kumar S Dispenzieri A Lacy M Hayman S Buadi F Colby C Revised prognostic staging system for light chain amyloidosis incorporating cardiac biomarkers and serum free light chain measurements. *J Clin Oncol.* (2012) 30:989–95. 10.1200/JCO.2011.38.5724 22331953 PMC3675680

[9] Palladini G Sachchithanantham S Milani P Gillmore J Foli A Lachmann H A European collaborative study of cyclophosphamide, bortezomib, and dexamethasone in upfront treatment of systemic AL amyloidosis. *Blood.* (2015) 126:612–5. 10.1182/blood-2015-01-620302 25987656

[10] Lilleness B Ruberg F Mussinelli R Doros G Sanchorawala V. Development and validation of a survival staging system incorporating BNP in patients with light chain amyloidosis. *Blood.* (2019) 133:215–23. 10.1182/blood-2018-06-858951 30333122

[11] Nicol M Baudet M Brun S Harel S Royer B Vignon M Diagnostic score of cardiac involvement in AL amyloidosis. *Eur Heart J Cardiovasc Imaging.* (2020) 21:542–8. 31292624 10.1093/ehjci/jez180

[12] Kim D Choi J Kim K Kim S Kim J Jeon E. Clinical and prognostic implications of capillary density in patients with cardiac light chain amyloidosis. *ESC Heart Fail.* (2021) 8:5594–9. 10.1002/ehf2.13604 34528755 PMC8712828

[13] Larsen B Mereuta O Dasari S Fayyaz A Theis J Vrana J Correlation of histomorphological pattern of cardiac amyloid deposition with amyloid type: a histological and proteomic analysis of 108 cases. *Histopathology.* (2016) 68:648–56. 10.1111/his.12793 26212778

[14] Medvedofsky D Kebed K Laffin L Stone J Addetia K Lang R Reproducibility and experience dependence of echocardiographic indices of left ventricular function: side-by-side comparison of global longitudinal strain and ejection fraction. *Echocardiography.* (2017) 34:365–70. 10.1111/echo.13446 28185312

[15] Singh A Carvalho Singulane C Miyoshi T Prado A Addetia K Bellino M Normal values of left atrial size and function and the impact of age: results of the world alliance societies of echocardiography study. *J Am Soc Echocardiogr.* (2022) 35:154–64.e3. 10.1016/j.echo.2021.08.008 34416309

[16] Kristen A Brokbals E Aus dem Siepen F Bauer R Hein S Aurich M Cardiac amyloid load: a prognostic and predictive biomarker in patients with light-chain amyloidosis. *J Am Coll Cardiol.* (2016) 68:13–24. 10.1016/j.jacc.2016.04.035 27364045

[17] Pucci A Aimo A Musetti V Barison A Vergaro G Genovesi D Amyloid deposits and fibrosis on left ventricular endomyocardial biopsy correlate with extracellular volume in cardiac amyloidosis. *J Am Heart Assoc.* (2021) 10:e020358. 10.1161/JAHA.120.020358 34622675 PMC8751897

[18] Koyama J Falk R. Prognostic significance of strain doppler imaging in light-chain amyloidosis. *JACC Cardiovasc Imaging.* (2010) 3:333–42. 10.1016/j.jcmg.2009.11.013 20394893

[19] Lei C Zhu X Hsi D Wang J Zuo L Ta S Predictors of cardiac involvement and survival in patients with primary systemic light-chain amyloidosis: roles of the clinical, chemical, and 3-D speckle tracking echocardiography parameters. *BMC Cardiovasc Disord.* (2021) 21:43. 10.1186/s12872-021-01856-3 33478398 PMC7819214

