# Supplementary material for: Evaluation of contemporary echocardiographic and histomorphology parameters in predicting mortality in patients with endomyocardial biopsy-proven cardiac AL amyloidosis
Source: Front Cardiovasc Med. 2023 Jan 24;9:1073804. doi: 10.3389/fcvm.2022.1073804 (PMC9902366; doi:10.3389/fcvm.2022.1073804)
Supplement: Supplementary file 1 [file Data_Sheet_1.docx]

Supplementary Table 1: Additional demographic, clinical and echocardiographic characteristics of 43 patients with endomyocardial biopsy-proven cardiac AL amyloidosis.

|  | Total N=43 |
| --- | --- |
| Age>65 years, n (%) | 24 (55.8) |
| BSA in m^2^, mean ± SD | 1.97±0.27 |
| Cerebrovascular accident, n (%) | 1 (2.3) |
| Chronic Lung Disease, n (%) | 4 (9.3) |
| **Baseline laboratory values:** |  |
| Hemoglobin in g/dL, mean ± SD | 11.6±1.9 |
| Platelets in K/uL, median (IQR) | 240.5 (165,306) |
| Absolute neutrophil count x1000/mm3, median (IQR) | 4.43 (2.96,6.4) |
| BNP in pg/mL, median (IQR) | 471.5(163,1408.5) |
| Troponin I in ng/mL, median (IQR) | 0.07(0.04,0.24) |
| **Echocardiographic characteristics:** |  |
| LVEDV in mL, mean ± SD | 104.78±37.80 |
| RWT, median (IQR) | 0.61 (0.48,0.75) |
| Lateral e’ velocity in cm/s, mean ± SD | 6.45±2.14 |
| Septal e’ velocity, mean ± SD | 5.47±1.91 |
| E/e’ average, median (IQR) | 12.91 (11.31,18.71) |
| MV E max velocity in cm/s, mean ± SD | 84.20±26.10 |
| MV A max velocity in cm/s, median (IQR) | 72.88 (54.59,88.85) |
| E/A, median (IQR) | 1.21 (0.72,1.7) |
| LAVI in mL/m^2^, mean ± SD | 30.79±10.31 |
| RV FAC in %, mean ± SD | 34.70±10.31 |
| RV S’ in cm/s, mean ± SD | 7.08±1.87 |
| RV TAPSE in cm, mean ± SD | 1.33±0.41 |
| LA strain in %, mean ± SD | 15.53±8.23 |
| RA strain in %, mean ± SD | 16.21±8.77 |

Supplementary Table 2: Association of clinical, echocardiographic and histomorphology characteristics with likelihood of mortality in the univariate Cox regression analysis.

|  | Univariate Cox regression on mortality (mortality event=29)  HR (95% CI) | p-value |
| --- | --- | --- |
| Age at biopsy | 1.015 (0.979-1.052) | 0.4128 |
| Age>65 years | 1.806 (0.838-3.892) | 0.1316 |
| Weight | 1.001 (0.984-1.019) | 0.8688 |
| BSA | 0.899 (0.239-3.373) | 0.8741 |
| Race: |  |  |
| White | 1.000 |  |
| Black | 1.107 (0.480-2.550) | 0.8114 |
| Other | 1.249 (0.365-4.274) | 0.7228 |
| Female sex | 1.719 (0.824-3.583) | 0.1486 |
| Hypertension | 0.636 (0.294-1.372) | 0.2483 |
| **Dyslipidemia** | **2.660 (1.252-5.649)** | **0.0109** |
| Diabetes mellitus | 1.888 (0.758-4.701) | 0.1723 |
| Coronary artery disease | 0.346 (0.082-1.467) | 0.1498 |
| End-stage renal disease on hemodialysis | 2.424 (0.726-8.090) | 0.1499 |
| Cerebrovascular accident | 5.502 (0.677-44.730) | 0.1107 |
| Peripheral arterial disease | 0.169 (0.010-2.985) | 0.2249 |
| Chronic Lung Disease | 0.927 (0.219-3.919) | 0.9177 |
| **Plasma Cell dyscrasia Therapies:** |  |  |
| **Stem cell transplantation*** | **0.213 (0.083-0.547)** | **0.0012** |
| SCT before endomyocardial biopsy | 0.631 (0.185-2.150) | 0.4619 |
| Proteasome inhibitors (eg bortezomib) | 1.311 (0.558-3.078) | 0.5343 |
| Alkylating Agents (eg cyclophosphamide) | 0.614 (0.293-1.284) | 0.1950 |
| Immunomodulators (eg lenalidomide) | 1.164 (0.537-2.527) | 0.7002 |
| Anti-CD38 monoclonal Abs (eg daratumumab) | 0.658 (0.196-2.203) | 0.4971 |
| **Rituximab** | **3.023 (1.003-9.110)** | **0.0494** |
| Elotuzumab | 0.694 (0.159-3.034) | 0.6278 |
| Vorinostat | 0.644 (0.084-4.916) | 0.6709 |
| **Baseline laboratory values:** |  |  |
| Hemoglobin | 0.945 (0.771-1.157) | 0.5811 |
| Hemoglobin <12 g/dL | 1.455 (0.678-3.121) | 0.3354 |
| Platelets | 1.000 (0.997-1.003) | 0.9590 |
| Platelets <150 K/uL | 0.667 (0.229-1.941) | 0.4573 |
| Absolute neutrophil count | 0.975 (0.923-1.030) | 0.3621 |
| **Absolute Neutrophils < 1.7 x1000/mm^3^** | **5.353 (1.102-26.002)** | **0.0375** |
| INR>1.1 | 1.023 (0.446-2.347) | 0.9563 |
| Creatinine | 1.068 (0.871-1.311) | 0.5264 |
| BNP | 1.000 (1.000-1.000) | 0.2378 |
| **Troponin I** | **8.379 (1.127-62.301)** | **0.0378** |
| Elevated natriuretic peptide levels (BNP or NT-proBNP) | 1.467 (0.345-6.247) | 0.6042 |
| Elevated troponin levels (I or T) | 2.082 (0.605-7.161) | 0.2447 |
| **Echocardiographic characteristics:** | HR (95% CI) | p-value |
| **LVEF** | **0.958 (0.923-0.995)** | **0.0257** |
| **LVEF<50%** | **2.894 (1.299-6.445)** | **0.0093** |
| LVEDV | 0.997 (0.988-1.007) | 0.5908 |
| LVEDVi | 0.995 (0.975-1.016) | 0.6652 |
| LVIDD | 0.875 (0.505-1.515) | 0.6339 |
| LVPWD | 1.567 (0.567-4.332) | 0.3866 |
| RWT | 1.829 (0.405-8.255) | 0.4323 |
| RWT>0.42 | 0.909 (0.309-2.678) | 0.8632 |
| LV mass | 1.001 (0.993-1.008) | 0.8923 |
| Lateral e’ velocity | 0.874 (0.701-1.091) | 0.2333 |
| Lateral e’ velocity <10 cm/s | 0.556 (0.128-2.414) | 0.4334 |
| Septal e’ velocity | 0.810 (0.625-1.050) | 0.1122 |
| Septal e’ velocity <7 cm/s | 1.679 (0.475-5.932) | 0.4207 |
| E/e’ average | 1.077 (0.997-1.163) | 0.0593 |
| E/e’ average >14 | 1.535 (0.643-3.665) | 0.3345 |
| MV E max velocity | 1.002 (0.983-1.021) | 0.8711 |
| MV A max velocity | 0.990 (0.969-1.012) | 0.3603 |
| E/A | 1.489 (0.670-3.309) | 0.3282 |
| LAVI | 1.014 (0.971-1.058) | 0.5298 |
| LAVI>34 mL/m^2^ | 1.205 (0.540-2.688) | 0.6485 |
| RV FAC | 0.989 (0.953-1.027) | 0.5667 |
| RV FAC<35% | 1.068 (0.484-2.356) | 0.8715 |
| RV GLS | 0.982 (0.929-1.037) | 0.5096 |
| RV S’ | 0.862 (0.688-1.081) | 0.1986 |
| RV S’ <9.5 cm/s | 2.489 (0.584-10.602) | 0.2175 |
| **RV TAPSE** | **0.299 (0.105-0.855)** | **0.0242** |
| RV TAPSE<1.6 cm | 2.026 (0.694-5.917) | 0.1964 |
| LV GLS | 1.137 (0.993-1.303) | 0.0633 |
| LV GLS > -18% | - | - |
| Apex GLS | 1.048 (0.962-1.141) | 0.2835 |
| **Mid LV GLS** | **1.141 (1.015-1.282)** | **0.0273** |
| Basal LV GLS | 1.080 (0.977-1.193) | 0.1327 |
| Circumferential LV strain | 1.073 (0.999-1.153) | 0.0533 |
| Circumferential LV strain > -23% | 2.437 (0.725-8.191) | 0.1497 |
| **Radial LV strain** | **0.935 (0.884-0.989)** | **0.0193** |
| Rad LV strain <21% | 4.406 (0.593-32.745) | 0.1473 |
| **LA GLS** | **0.902 (0.845-0.962)** | **0.0019** |
| **LA GLS <13.5%** | **5.394 (2.072-14.041)** | **0.0006** |
| **RA GLS** | **0.952 (0.909-0.996)** | **0.0346** |
| **RA GLS <14.5%** | **2.711 (1.209-6.080)** | **0.0155** |
| **Histologic characteristics:** |  |  |
| Interstitial deposit >5% of myocardial area | 1.252 (0.590-2.659) | 0.5583 |
| Pattern of interstitial deposit: |  |  |
| - Diffuse pericellular | 1.552 (0.579-4.156) | 0.3821 |
| - Discrete pericellular, nodular, mixed | 1.159 (0.400-3.358) | 0.7854 |
| Vascular deposit | 0.878 (0.387-1.992) | 0.7560 |
| **Staging systems:** |  |  |
| Mayo 2004 staging   - II - III | 1.000  1.097 (0.226-5.325) | 0.9086 |
| Mayo 2012 staging   - I-II - III - IV | 1.000  0.636 (0.056-7.262)  4.037 (0.348-46.856) | 0.7155  0.2646 |
| Boston University staging   - I-II - IIIA-IIIB | 1.000  2.608 (0.813-8.372) | 0.1072 |
| Composite staging   - Early stages (I-II by Mayo or Boston) - Advanced stages (III-IV by Mayo or Boston) | 1.000  1.945 (0.712-5.311) | 0.1942 |

* SCT as a time-varying covariate

Supplementary Table 3: Association of demographic, baseline clinical and echocardiographic characteristics with interstitial amyloid deposit ≥5% of myocardial area on biopsy samples.

|  | Univariate logistic regression on Interst. deposit >5%  (Interst. deposit >5% event=26) OR (95% CI) | p-value |
| --- | --- | --- |
| Age at biopsy | 0.989 (0.936-1.046) | 0.7050 |
| Age>65 years, n (%) | 0.817 (0.237-2.811) | 0.7481 |
| Weight | 1.014 (0.982-1.048) | 0.3834 |
| BSA | 4.197 (0.354-49.801) | 0.2558 |
| Race: |  |  |
| - White | 1.000 |  |
| - Black | 0.421 (0.104-1.709) | 0.2263 |
| - Other | 0.14 (0.013-1.562) | 0.1102 |
| Female sex | 0.825 (0.241-2.822) | 0.7592 |
| Hypertension | 1.481 (0.396-5.538) | 0.5599 |
| Dyslipidemia | 1.344 (0.380-4.753) | 0.6460 |
| Diabetes mellitus | 13.464 (0.589-307.903) | 0.1035 |
| Coronary artery disease | 0.13 (0.013-1.286) | 0.0810 |
| End-stage renal disease on hemodialysis | 1.333 (0.111-15.960) | 0.8203 |
| Cerebrovascular accident | 2.143 (0.021-216.115) | 0.7461 |
| Peripheral arterial disease | 0.64 (0.037-10.975) | 0.7582 |
| Chronic Lung Disease | 6.996 (0.251-194.797) | 0.2517 |
| **Plasma Cell dyscrasia Therapies:** |  |  |
| SCT prior to cardiac amyloidosis | 0.389 (0.058-2.618) | 0.3317 |
| Proteasome inhibitors (eg bortezomib) | 2.94 (0.745-11.599) | 0.1236 |
| Alkylating Agents (eg cyclophosphamide) | 1.8 (0.522-6.204) | 0.3519 |
| Immunomodulators (eg lenalidomide) | 0.429 (0.114-1.618) | 0.2113 |
| Anti-CD38 monoclonal Abs (eg daratumumab) | 0.187 (0.018-1.969) | 0.1627 |
| Rituximab | 0.625 (0.079-4.920) | 0.6553 |
| Elotuzumab | 0.117 (0.003-5.088) | 0.2649 |
| Vorinostat | 0.21 (0.002-19.984) | 0.5022 |
| **Baseline laboratory values:** |  |  |
| Hemoglobin | 1.095 (0.768-1.561) | 0.6157 |
| Hemoglobin <12 g/dL | 0.629 (0.162-2.445) | 0.5036 |
| Platelets | 1.003 (0.998-1.008) | 0.2818 |
| Platelets <150 K/uL | 1.212 (0.246-5.969) | 0.8129 |
| Absolute neutrophil count | 1.054 (0.947-1.174) | 0.3362 |
| Absolute Neutrophils < 1.7 x1000/mm^3^ | 4.657 (0.105-207.116) | 0.4269 |
| INR>1.1 | 0.489 (0.114-2.097) | 0.3355 |
| Creatinine | 0.974 (0.613-1.549) | 0.9118 |
| BNP | 1 (1.000-1.001) | 0.4323 |
| Troponin I | 0.91 (0.030-27.348) | 0.9565 |
| Elevated natriuretic peptide levels (BNP or NT-proBNP) | 0.697 (0.065-7.490) | 0.7658 |
| Elevated troponin levels (I or T) | 2.251 (0.388-13.069) | 0.3661 |
| **Echocardiographic characteristics:** |  |  |
| LVEF | 1 (0.938-1.067) | 0.9925 |
| LVEF<50% | 1.833 (0.475-7.070) | 0.3788 |
| LVEDV | 0.996 (0.979-1.014) | 0.6826 |
| LVEDVi | 0.982 (0.944-1.021) | 0.3647 |
| LVIDD | 0.48 (0.177-1.301) | 0.1490 |
| LVPWD | 10.625 (0.850-132.828) | 0.0667 |
| RWT | 35.593 (0.790-1603.71) | 0.0660 |
| RWT>0.42 | 25.662 (0.990-665.065) | 0.0507 |
| LV mass | 1.014 (1.000-1.029) | 0.0570 |
| LV mass >195.7g ^ | 4.583 (1.117-18.802) | 0.0345 |
| Lateral e’ velocity | 0.916 (0.653-1.285) | 0.6112 |
| Lateral e’ velocity <10 cm/s | 1.727 (0.098-30.450) | 0.7089 |
| Septal e’ velocity | 0.685 (0.450-1.043) | 0.0775 |
| Septal e’ velocity <7 cm/s | 4.499 (0.679-29.796) | 0.1190 |
| E/e’ average | 1.072 (0.934-1.231) | 0.3202 |
| E/e’ average >14 | 0.9 (0.212-3.822) | 0.8865 |
| MV E max velocity | 0.993 (0.967-1.021) | 0.6293 |
| MV A max velocity | 0.982 (0.950-1.014) | 0.2617 |
| E/A | 1.77 (0.495-6.325) | 0.3798 |
| LAVI | 1.006 (0.941-1.075) | 0.8638 |
| LAVI>34 mL/m^2^ | 1.467 (0.376-5.723) | 0.5814 |
| RV FAC | 1.019 (0.955-1.089) | 0.5654 |
| RV FAC<35% | 0.791 (0.211-2.972) | 0.7287 |
| RV GLS | 0.966 (0.877-1.064) | 0.4822 |
| RV S’ | 0.935 (0.656-1.334) | 0.7122 |
| RV S’ <9.5 cm/s | 2.5 (0.364-17.172) | 0.3514 |
| RV TAPSE | 0.379 (0.069-2.074) | 0.2633 |
| **RV TAPSE<1.6 cm ^** | **8.75 (1.487-51.499)** | **0.0165** |
| LV GLS | 1.184 (0.944-1.485) | 0.1431 |
| Apex GLS | 1.006 (0.872-1.160) | 0.9373 |
| Mid LV GLS | 1.158 (0.945-1.419) | 0.1560 |
| Basal LV GLS | 1.114 (0.952-1.304) | 0.1773 |
| Circumferential LV strain | 0.959 (0.851-1.080) | 0.4886 |
| Circumferential LV strain >-23% | 1.309 (0.288-5.948) | 0.7273 |
| Radial LV strain | 1.027 (0.950-1.111) | 0.4989 |
| Rad LV strain <21% | 0.339 (0.034-3.376) | 0.3565 |
| LA GLS | 1.042 (0.958-1.134) | 0.3341 |
| LA GLS<13.5 | 1.247 (0.339-4.589) | 0.7401 |
| RA GLS | 0.943 (0.871-1.021) | 0.1476 |
| RA GLS<14.5 | 1.95 (0.520-7.311) | 0.3220 |

^ Statistically significant in the multivariate logistic regression analysis: RV TAPSE: OR 8.1, 95% CI 1.2-54.3 for RV TAPSE<1.6cm vs ≥1.6cm, p=0.032. LV mass: OR 5.5, 95% CI 1.1-26.8 for >195.7g vs ≤195.7g, p=0.036.
